# Real-World Use and Outcomes of ALK-Positive Crizotinib-Treated Metastatic NSCLC in US Community Oncology Practices: A Retrospective Observational Study

**DOI:** 10.3390/jcm7060129

**Published:** 2018-05-29

**Authors:** Craig Reynolds, Elizabeth T. Masters, Jenny Black-Shinn, Marley Boyd, Jack Mardekian, Janet L. Espirito, Marc Chioda

**Affiliations:** 1 Florida Cancer Specialists and Research Institute, Ocala, FL 34471, USA; chrmd@hotmail.com; 2 The US Oncology Network/McKesson Specialty Health, The Woodlands, TX 77380, USA; jblackshinn15@gmail.com (J.B.-S.); marley.boyd@mckesson.com (M.B.); 3 Pfizer Oncology, New York, NY 10017, USA; elizabeth.masters@pfizer.com (E.T.M.); jack.mardekian@pfizer.com (J.M.); marcd.chioda@pfizer.com (M.C.)

**Keywords:** crizotinib, real-world utilization, patient outcomes, advanced NSCLC, ALK-positive

## Abstract

Introduction: Around 3–5% of non-small cell lung cancers (NSCLC) are ALK-positive. Crizotinib was the first approved ALK inhibitor from clinical trials. However, there are less data on the utilization and patient outcomes associated with crizotinib in real-world clinical practice. Methods: This was a retrospective, observational study of adult crizotinib-treated ALK-positive metastatic NSCLC patients who received treatment between 1 September 2011 and 31 October 2014, with follow up through 31 December 2015. Data were obtained via programmatic queries of the US Oncology Network/McKesson Specialty Health electronic health record database, supplemented with chart abstraction. Overall survival (OS) and time to treatment failure (TTF) were estimated from crizotinib initiation using the Kaplan–Meier (KM) method. Results: Of the *n* = 199 ALK-positive crizotinib-treated patients meeting eligibility criteria, crizotinib was prescribed as first line (1 L) in *n* = 123 (61.8%). The majority (88.9%) had confirmed adenocarcinoma histology and 32.2% had brain metastases at initial diagnosis. Median age at crizotinib initiation was 60.2 years (range 27.1–88.2); 54.8% were never smokers, 33.7% were former smokers. Treatment of 250 mg, twice daily, was most commonly prescribed (89.5%) with the dose unchanged from an initial dose in 79.4% of patients. The primary discontinuation reason was progression (*n* = 91, 58.7%). Patients (3.2%) were identified as discontinuing crizotinib as a result of treatment-related toxicity. With median follow-up time of 13.0 months (min–max = 0.03–46.6), median OS from crizotinib initiation was 33.8 months (95% CI = 24.3–38.8). Median TTF was 10.4 months. Conclusions: Crizotinib usage evaluated within the real-world setting is consistent with prior phase III clinical trial data, and illustrates the real-world effectiveness of crizotinib.

## 1. Introduction

Biomarker driven therapies have revolutionized the treatment of non-small cell lung cancer (NSCLC). A genetic alteration of the anaplastic lymphoma kinase (ALK) gene is present in 3–5% of NSCLCs [1,2,3,4,5,6,7,8]. The first FDA-approved ALK inhibitor for ALK-positive metastatic NSCLC treatment in 2011, crizotinib, has shown significant improvement in progression-free survival (PFS) and tumor responses in patients with metastatic NSCLC who carry the ALK gene rearrangement [9]. Crizotinib is an oral ATP competitive selective inhibitor of the ALK, MET, and ROS-1 tyrosine kinases that inhibit tyrosine phosphorylation of activated ALK at nanomolar concentrations [10,11]. While crizotinib has demonstrated significant improvement in PFS in phase III studies [12,13], there are less data on the utilization and patient outcomes associated with crizotinib in real-world clinical practice. Our study sought to examine treatment patterns and outcomes of crizotinib utilization in NSCLC patients from U.S. community oncology practices, and to explore the impact of line of therapy and presence of brain metastases on outcomes.

## 2. Methods

Institutional review board approval was received for this retrospective study of patients diagnosed with metastatic NSCLC and treated with crizotinib in The U.S. Oncology Network (USON) of community-based oncology practices, utilizing the iKnowMed™ (iKM) electronic health record (EHR). iKM is an integrated web-based database and oncology-specific EHR system maintained by McKesson Specialty Health (MSH), that captures outpatient practice encounter histories from approximately 1500 community-based oncology providers across practices in 19 states. The patient identification period consisted of patients initiating crizotinib treatment between 1 September 2011 through 31 October 2014, with follow-up data collected through 31 December 2015. The study time period was selected to capture crizotinib-treated patients post FDA approval, and to ensure sufficient follow-up, including survival outcomes. This study reflects the time during which crizotinib was the only approved first-line agent for ALK-positive patients.

Data for this study was obtained from the iKM database, supplemented with electronic medical chart abstraction, claims data, and vital status from Social Security Death Index (SSDI). Records, including diagnosis date, age, sex, race, practice region, histology, sites of metastases, performance status, tumor markers, comorbidities, and smoking history, were reviewed. Treatment records, including crizotinib doses, treatment dates, and line of therapy were identified.

Duration of therapy, healthcare resource use, and clinical outcomes, including overall survival (OS), and time to treatment failure (TTF), were assessed. OS was defined as the time from initiation of crizotinib until death from any cause. TTF was defined as time from initiation of crizotinib until discontinuation of treatment for any reason (e.g., progression, toxicity, other).

Pre-crizotinib treatment was identified as those regimens given within 30 days prior to the first crizotinib prescription date. Post-crizotinib treatment was identified as those regimens given within 30 days after the last crizotinib prescription date. First line (1 L) crizotinib treatment was defined as the first identified treatment with crizotinib not preceded by any other treatment for NSCLC therapy. Second line or later (≥2 L) crizotinib treatment was defined as treatment with crizotinib that was preceded by one or more systemic chemotherapeutic regimens for NSCLC.

Healthcare resource utilization that occurred during crizotinib treatment (crizotinib initiation through 30 days after the last identified treatment of crizotinib therapy) was captured utilizing CPT codes, and summarized for the specific categories of healthcare usage (e.g., outpatient visits, laboratory procedures, radiotherapy, imaging).

The cost from claims of each healthcare use was estimated based on the amount allowed to the rendering service provider on the payer remittance per the 2014 Medicare data. These amounts are normalized costs for the treatment of these patient subgroups treated with the MSH/USON community oncology practices. Healthcare expenditure was reported by health resource category for patients overall, and further calculated as per patient per month cost.

Patients were included if they were ≥18 years at initial NSCLC diagnosis, had evidence of metastatic disease, initiated treatment with crizotinib, and received care at an USON practice with full iKM EHR capabilities over the study period. Patients with a concomitant diagnosis of other primary cancers during the study period or enrolled in clinical trials prior to crizotinib initiation were excluded.

### Statistical Methods

Patient demographic, clinical, and treatment characteristics were summarized. The association between patient characteristics and line of therapy (LOT), presence or absence of brain metastases, were compared with Fisher’s exact test (for the categorical variables) and Kruskal–Wallis test (for the continuous variables), as applicable. Survival and time to treatment failure were calculated from the date of initiation (prescription date) of crizotinib with the Kaplan–Meier (KM) method. *p*-Values comparing curves were calculated with log-rank tests.

## 3. Results

### 3.1. Demographics and Baseline Characteristics

A total of 70,300 patients with NSCLC were identified during the study period; 274 metastatic patients initiated treatment with crizotinib during the study period, and 212 met all eligibility criteria. Of those, 199 had a confirmed documented ALK-positive statu.

The majority of physician practices were located in the south (55.8%) followed by the west (27.1%; Table 1). The median age at crizotinib initiation was 60.2 years (min–max 27.1–88.2); 54.8% were never smokers, 33.7% were former smokers, and 77.4% had an Eastern Cooperative Oncology Group (ECOG) performance status score of 0 or 1. Sites of distant metastases were mostly bone (45.7%) followed by distant lymph nodes (36.7%), brain (32.2%), and liver (24.6%). The majority of patients (88.9%) had confirmed adenocarcinoma histology at initial NSCLC diagnosis.

### 3.2. Crizotinib Treatment Patterns

Crizotinib was prescribed as 1 L treatment in 61.8% (*n* = 123) and ≥2 L in 38.2% (*n* = 76) of patients (Table 2). Total average duration of crizotinib treatment was 11.5 months (SD = 10.6). Treatment of 250 mg, twice daily, was most commonly prescribed (89.5%) at initial treatment in the overall population, and was consistent by LOT and presence or absence of brain metastases. Dose remained unchanged from the initial dose in 79.4% of all NSCLC patients evaluated.

Dose reductions occurred in 13.1% of patients. Radiotherapy was given during crizotinib treatment in 18.6% of patients. Patients with brain metastases were more likely to receive radiotherapy compared to those without brain metastases (35.5% versus 10.4%, *p* < 0.0001). The most common treatment received prior to the start of crizotinib was platinum doublet (32.7%).

The most common primary discontinuation reason was progression (58.7%) followed by death (16.8%); and 3.2% of patients were identified as discontinuing crizotinib as a result of treatment-related toxicity. Twenty-two percent of patients in this study were identified as ongoing crizotinib therapy at the end of the study period and 39.2% of the overall population moved on to a new therapy after crizotinib. Within those patients receiving post crizotinib treatment, the most common treatment received was ceritinib (40.9%).

### 3.3. Crizotinib Clinical Outcomes

**Figure 1:**
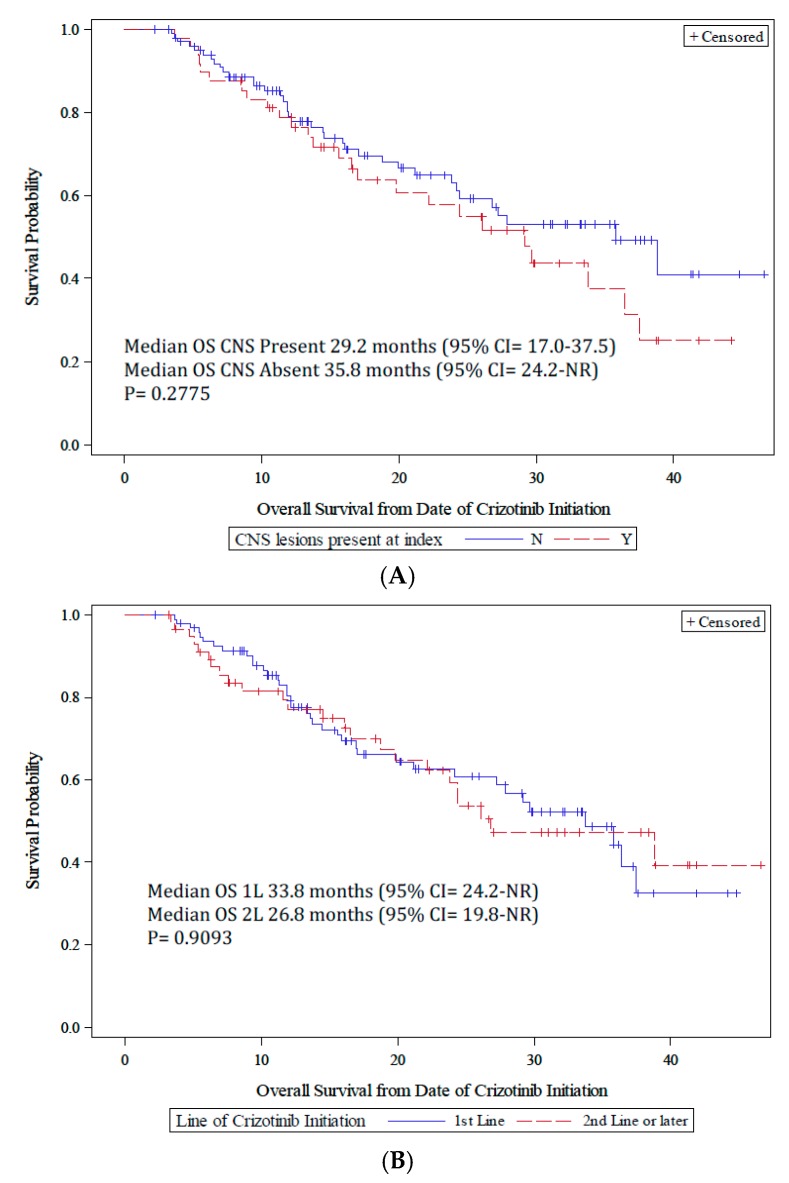
(**A**) Kaplan–Meier curve of overall survival from crizotinib initiation by CNS metastases; (**B**) Kaplan–Meier curve of overall survival from crizotinib initiation by line of therapy (LOT).

**Figure 2:**
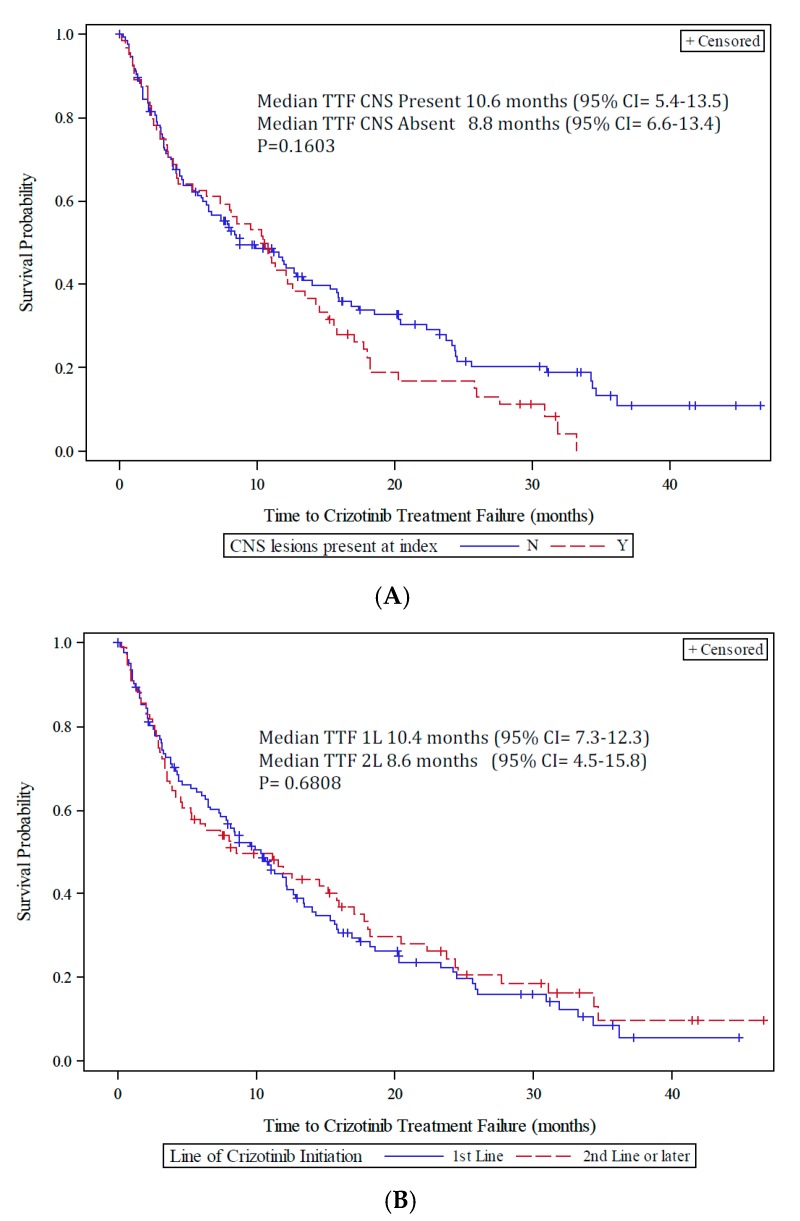
(**A**) Kaplan–Meier curve of time to treatment failure from crizotinib initiation by CNS metastases; (**B**) Kaplan–Meier curve of time to treatment failure from crizotinib initiation by LOT.

Patients were observed for a median of 13.0 months (min–max = 0.03, 46.6). The proportion of patients with <3 months of crizotinib treatment was 24.6% and 75.4% for patients with ≥3 months of treatment (Table 2). Median survival in patients treated for at least 3 months was 33.8 months, with 1- and 2-year survival probabilities of 79.0% (95% CI: 71.2, 84.9) and 61.3% (95% CI: 51.8, 69.4), respectively. Although overall survival time was numerically reduced in patients initiating crizotinib as 2 L or greater and in patients with CNS lesions present before crizotinib initiation, it was not statistically different (*p* = 0.91 for crizotinib LOT and *p* = 0.28 by brain metastases; Figure 1A,B). Median TTF was 10.4 months (95% CI: 7.4, 12.2) in the overall population, and was similar by LOT (*p* = 0.68) and brain metastases (*p* = 0.16; Figure 2A,B). Median time to treatment failure was 10.4 months (95% CI = 7.3, 12.3 months) in the patients initiating crizotinib as 1 L and 8.6 months (95% CI = 4.5, 15.8 months) for patients initiating crizotinib as 2 L or later.

### 3.4. Healthcare Resource Utilization and Cost

Claims data during crizotinib treatment was available for 199 patients. Table 3 presents information on healthcare resource use and associated cost overall and by LOT during crizotinib treatment.

Sixty-one (30.7%) patients had at least one hospitalization identified from chart review during crizotinib treatment. One hundred fifty-three (76.9%) patients had a minimum of one laboratory related claim. Prevalence of at least one claim for radiotherapy and imaging in the overall population during crizotinib treatment consisted of *n* = 37 (18.6%) and *n* = 53 (26.6%), respectively. There were no statistically significant differences by crizotinib LOT for categories of healthcare use.

## 4. Discussion

This study examined the real-world treatment patterns and outcomes of patients with metastatic NSCLC treated with crizotinib in a U.S. community oncology setting. Broader real-world effectiveness, safety, and cost data may aid in improving quality and delivery of care, as well as outcomes, by accelerating current understanding of how best to incorporate treatments into everyday clinical practice.

Use of EHR data from a large network of practices allowed for increased sample size of a relatively less common type of NSCLC.

A total of 199 ALK-positive patients receiving crizotinib were included in the study. While the estimated prevalence of ALK-positive NSCLC is approximately 3–5%, only approximately 10% of those expected to be ALK-positive were identified and treated during the study period. The study period reflected the time frame immediately following approval of crizotinib as the first ALK-inhibitor, so not all patients may have been tested, or could have been tested for the ALK gene rearrangement, if there was insufficient tissue or if patients were not candidates for re-biopsy. However, it does raise the importance of education and awareness of biomarker testing. As the emergence of the benefit of ALK targeted therapy, and new second generation ALK inhibitors have emerged, we would anticipate this number to increase over time.

Consistent with prior studies [12,13], our population was predominately female (52.3%), never (54.8%) or former smokers (33.7%), with a greater prevalence of adenocarcinoma histology (88.9%). Our population was on average older (60 vs 51–52 years) than what has been reported by other studies [12,13].

The most common reason for treatment discontinuation in this population was disease progression, however, the study by Solomon et al. [13] documented that 73% of patients with progressive disease continued to receive crizotinib as 1 L beyond Response Evaluation Criteria in Solid Tumors (RECIST) defined progression.

Additionally, treatment appeared to be well tolerated, as 3.2% of patients discontinued as a result of treatment-related toxicity. This was similar to the results from Solomon et al. [13], as patients treated with crizotinib in that study tended to have low grade toxic events (grades 1–2) and 12% of patients permanently discontinued crizotinib treatment resulting from adverse events with varying etiology.

With a median follow-up time of 13.0 months, median OS from crizotinib initiation in patients treated with crizotinib for at least 3 months was 33.8 months with 1- and 2-year survival rates of 79.0% and 61.3%, and did not differ by presence of brain metastases or LOT. Overall survival was calculated for patients treated with crizotinib for a minimum of 3 months’ duration, to adequately assess the effectiveness of crizotinib treatment. Relative to a previously published phase III clinical trial [12], this study had a longer follow-up, providing further evidence of crizotinib treatment real-world effectiveness for metastatic NSCLC. Prior studies evaluating survival identified estimates for OS as 21.7 months in second-line treatment in one study, while median OS was not reached in another first-line phase III study [13,14].

Overall, our population had a survival rate of 79.4% at 1 year, and 61.6% at 2 years. These estimates did not differ by the strata of interest, and are consistent with estimates from another phase III study [13].

Sixty-one (30.7%) patients in the overall population had at least one hospitalization identified during chart review related to NSCLC during crizotinib treatment. A study conducted by Karve et al. [15] assessing health resource utilization in metastatic NSCLC patients, using administrative claims data, found that 44.9% of NSCLC patients had at least one hospital admission claim related to NSCLC. The major difference between the studies is the current study specifically evaluated hospital admissions related to NSCLC during crizotinib treatment, whereas the study conducted by Karve et al. [15] evaluated admission claims related to NSCLC which encompassed greater variability in treatment patterns.

### Study Limitations

Limitations include the retrospective nature of this study, and potential for documentation bias if there were errors or omissions in the medical record. The iKM system is used for clinical practice reasons, not solely for research purposes, which may impede the standardization of the data collection methods and instruments. Not all community oncology practices are included in the iKM dataset, and not all of the U.S. Oncology clinics utilize iKM. The study population consists of patients solely treated within the USON network, and these patients may not represent the general patient population with metastatic NSCLC outside the network. As crizotinib is an oral medication, adherence and precise treatment start and stop dates may not be recorded in the EHR and duration of treatment is assessed as best captured in the records.

## 5. Conclusions

Crizotinib usage evaluated within the real-world setting is consistent with prior phase III crizotinib clinical trial data illustrating the treatment effectiveness of crizotinib. The lack of deviation from the initial starting dose suggests treatment tolerability.

## Figures and Tables

**Table 1:** Demographics and baseline characteristics.

Characteristic	All Patients (*n* = 199)
**Females**	104 (52.3)
**Age at crizotinib initiation, years**	
Median (min, max)	60.2 (27.1–88.2)
18–35	8 (4.0)
36–45	26 (13.1)
46–55	38 (19.1)
56–65	59 (29.7)
>65	68 (34.2)
**BMI, kg/m^2^**	
Median (min, max)	25.6 (15.9–50.3)
**Census regions (physicians), *n* (%)**	
South	111 (55.8)
West	54 (27.1)
Midwest	24 (12.1)
Northeast	10 (5.0)
**ECOG PS at advanced NSCLC diagnosis**	
0	22 (11.1)
1	132 (66.3)
2	27 (13.6)
3	1 (0.5)
Unknown	17 (8.5)
**Stage at initial NSCLC diagnosis**	
Early (stage IA, IB, IIA, IIB)	16 (8.0)
Limited/regional (stage IIIA)	15 (7.5)
Locally advanced (stage IIIB)	22 (11.1)
Metastatic (stage IV)	133 (66.8)
Missing/unknown	13 (6.5)
**Histology**	
Adenocarcinoma (mixed or not)	177 (88.9)
Squamous	4 (2.0)
Not otherwise specified (NOS)	1 (0.50)
Missing/unknown	17 (8.5)
**Smokingstatus**	
Current	23 (11.6)
Former	67 (33.7)
Never	109 (54.8)
**Sites of metastases**	
Adrenal gland	18 (9.1)
Bone	91 (45.7)
Brain	64 (32.2)
Distant lymph nodes	73 (36.7)
Liver	49 (24.6)
Other ^1^	120 (60.3)

^1^ Other consists of identification of categories not previously specified (e.g., pleural cavity).

**Table 2:** Demographics, baseline characteristics and treatment patterns by crizotinib line of therapy and presence/absence of central nervous system (CNS) metastases.

		Line of Therapy for Crizotinib Initiation			CNS Metastases		
		First-Line (*n* = 123)	Second/Later-Line (*n* = 76)	*p*-Value	Present (*n* = 64)	Absent (*n* = 135)	*p*-Value
**Sex, *n* (%)**							
Female		63 (51.2)	41 (54.0)	0.77	34 (53.1)	70 (51.9)	0.88
**Age (years) at crizotinib initiation**							
Median (min, max)		59.3 (27.1, 88.2)	63.2 (28.3, 86.7)		59.4 (28.3 81.3)	61.5 (27.1, 88.2)	
**Age distribution (years)**							
18–35		3 (2.4)	5 (6.6)	0.08	3 (4.7)	5 (3.7)	0.47
36–45		19 (15.5)	7 (9.2)		11 (17.2)	15 (11.1)	
46–55		29 (23.6)	9 (11.8)		12 (18.8)	26 (19.3)	
56–65		33 (26.8)	26 (34.2)		19 (29.7)	40 (29.6)	
>65		39 (31.7)	29 (38.2)		19 (29.7)	49 (36.3)	
**Census regions (physicians), *n* (%)**							
Midwest		17 (13.8)	7 (9.2)	0.42	8 (12.5)	16 (11.9)	0.58
Northeast		4 (3.3)	6 (7.9)		2 (3.1)	8(5.9)	
South		69 (56.1)	42 (55.3)		33 (51.6)	78 (57.8)	
West		33 (26.8)	21 (27.6)		21 (32.8)	33 (24.4)	
**BMI**							
Median (min, max)		25.7 (15.9, 50.3)	24.9 (17.3, 49.7)		25.8 (17.3, 50.3)	25.2 (15.9,49.7)	
**ECOG at crizotinib initiation, *n* (%)**							
0		17 (13.8)	5 (6.6)	0.23	6 (9.4)	16 (11.9)	0.23
1		76 (61.8)	56 (73.7)		39 (60.9)	93 (68.9)	
2		18 (14.6)	9 (11.8)		13 (20.3)	14 (10.4)	
3		1 (0.8)	0		0 (0.0)	1 (0.7)	
Unknown		11 (8.9)	6 (7.9)		6 (9.4)	11 (8.2)	
**Disease stage at initial NSCLC diagnosis, *n* (%)**							
Early (stage IA, IB, IIA, IIB)		7 (5.7)	9 (11.8)	<0.01	2 (3.1)	14 (10.4)	<0.01
Limited/regional (stage IIIA)		6 (4.9)	9 (11.8)		6 (9.4)	9 (6.7)	
Locally advanced (stage IIIB)		8 (6.5)	14 (18.4)		2 (3.1)	20 (14.8)	
Metastatic (stage IV)		94 (76.4)	39 (51.3)		52 (81.3)	81 (60.0)	
Unknown		8 (6.5)	5 (6.6)		2 (3.1)	11 (8.2)	
**Histology at crizotinib initiation (%)**							
Adenocarcinoma (mixed or not)		113 (91.9)	64 (84.2)	0.42	57 (89.1)	120 (88.9)	0.11
Squamous		2 (1.6)	2 (2.6)		3 (4.7)	1 (0.7)	
Not otherwise specified (NOS)		0 (0.0)	1 (1.3)		0 (0.0)	1 (0.7)	
Missing/Unknown		8 (6.5)	9 (11.8)		4 (6.3)	13 (9.6)	
**Smokingstatus (closest to crizotinib initiation), *n* (%)**							
Current smoker		11 (8.9)	12 (15.8)	0.30	7 (10.9)	16 (11.9)	0.31
Former smoker		41 (33.3)	26 (34.2)		17 (26.6)	50 (37.0)	
Never smoked		71 (57.7)	38 (50.0)		40 (62.5)	69 (51.1)	
**Site(s) of distant metastases at crizotinib initiation, *n* (%)**							
Adrenal gland		11 (8.9)	7 (9.2)	1.00	6 (9.4)	12 (8.9)	1.00
Bone		56 (45.5)	35 (46.1)	1.00	31 (48.4)	60 (44.4)	0.65
Brain		43 (35.0)	21 (27.6)	0.35	64 (100.0)	0(0.0)	<0.01
Distant lymph nodes		46 (37.4)	27 (35.5)	0.88	16 (25.0)	57 (42.2)	0.02
Liver		34 (27.6)	15 (19.7)	0.24	18 (28.1)	31 (23.0)	0.48
Other		75 (61.0)	45 (59.2)	0.88	35 (54.7)	85 (63.0)	0.28
**Treatment Patterns**							
	**Overall (*n* = 199)**	**Line of Therapy for Crizotinib Initiation**		***p*-Value**	**CNS Metastases**		
		**First-Line (*n* = 123)**	**Second/Later-Line (*n* = 76)**		**Present (*n* = 64)**	**Absent (*n* = 135)**	***p*-Value**
**Total duration of crizotinib treatment**							
Mean (SD), months	11.5 (10.6)	11.0 (9.9)	12.3 (11.6)	0.66	11.4 (9.4)	11.5 (11.1)	0.64
Median (range), months	8.5 (0.2–48.3)	8.5 (0.2–46.6)	8.4 (0.3–48.3)		10.5 (0.2–33.2)	7.9 (0.3–48.3)	
**Total duration of crizotinib treatment, *n* (%)**							
<3 months	49 (24.6)	30 (24.4)	19 (25.0)	1.00	16 (25.0)	33 (24.4)	1.00
≥3 months	150 (75.4)	93 (75.6)	57 (75.0)		48 (75.0)	102 (75.6)	
**Cancer treatment received within 30 days before crizotinib start date, *n* (%) ^†^**	52 (26.1)	26 (21.1)	26 (34.2)	0.03	13 (20.3)	39 (28.9)	0.94
Platinum doublet ^1,±^	17 (32.7)	13 (50.0)	4 (15.4)		5 (38.5)	12 (30.8)	
Platinum triplet ^2,±^	13 (25.0)	7 (26.9)	6 (23.1)		7 (53.8)	6 (15.4)	
Pemetrexed ^±^	8 (15.4)	1 (3.8)	7 (26.9)		3 (23.1)	5 (12.8)	
Erlotinib ^±^	3 (5.8)	1 (3.8)	2 (7.7)		0 (0.0)	3 (7.7)	
Bevacizumab ^±^	2 (3.9)	0 (0.0)	2 (7.7)		0 (0.0)	2 (5.1)	
Other ^3,±^	9 (17.3)	4 (15.4)	5 (19.2)		2 (15.4)	7 (17.9)	
**Cancer treatment received within 30 days post crizotinib end date, *n* (%) ^†^**	71 (35.7)	50 (40.7)	21 (27.6)	0.21	24 (37.5)	47 (34.8)	0.99
Platinum doublet ^4,±^	16 (22.5)	12 (24.0)	4 (19.0)		6 (25.0)	10 (21.3)	
Platinum triplet ^5,±^	2 (2.8)	2 (4.0)	0 (0.0)		0 (0.0)	2 (4.3)	
Ceritinib ^±^	29 (40.9)	22 (44.0)	7 (33.3)		10 (41.7)	19 (40.4)	
Pemetrexed ^±^	5 (7.0)	2 (4.0)	3 (14.3)		2 (8.3)	3 (6.4)	
Alectinib ^±^	2 (2.8)	2 (4.0)	0 (0.0)		0 (0.0)	2 (4.3)	
Docetaxel ^±^	2 (2.8)	0 (0.0)	2 (9.5)		1 (4.2)	1 (2.1)	
Other ^6,±^	15 (21.1)	10 (20.0)	5 (23.8)		5 (20.8)	10 (21.3)	
**Initial crizotinib total daily dose, *n* (%)**							
250 mg QD	11 (5.5)	5 (4.1)	6 (7.9)	0.04	3 (4.7)	8 (5.9)	1.0
200 mg BID	10 (5.0)	3 (2.4)	7 (9.21)		3 (4.7)	7 (5.2)	
250 mg BID	178 (89.5)	115 (93.5)	63 (82.9)		58 (90.6)	120 (88.9)	
**Crizotinib total daily dose changes, *n* (%)**							
≥1 dose escalation	3 (1.5)	1 (0.8)	2 (2.6)	0.77	0 (0.0)	3 (2.2)	0.67
≥1 dose reduction	26 (13.1)	16 (13.0)	10 (13.2)		7 (10.9)	19 (14.1)	
≥1 dose reduction and ≥1 dose escalation	12 (6.0)	7 (5.7)	5 (6.6)		3 (4.7)	9 (6.7)	
No changes	158 (79.4)	99 (80.5)	59 (77.6)		54 (84.4)	104 (77.0)	
**Other cancer treatment during active crizotinib treatment, *n* (%)**							
Radiotherapy	37 (18.6)	24 (19.5)	13 (17.1)	0.71	23 (35.9)	14 (10.4)	<0.01
Other	49 (24.6)	33 (26.8)	16 (21.1)	0.40	22 (34.4)	27 (20.0)	0.03
**Primary reason(s) for final d/c of crizotinib ^7^, *n* (%)**							
Death	26 (16.8)	16 (16.7)	10 (17.0)	0.79	9 (15.8)	17 (17.4)	0.86
Disease progression	91 (58.7)	60 (62.5)	31 (52.5)		33 (57.9)	58 (59.2)	
Treatment-related toxicity or side effects	5 (3.2)	2 (2.1)	3 (5.1)		1 (1.8)	4 (4.1)	
Physician preference	6 (3.9)	3 (3.1)	3 (5.1)		3 (5.3)	3 (3.1)	
Patient preference	8 (5.2)	4 (4.2)	4 (6.8)		3 (5.3)	5 (5.1)	
Cost	1 (0.7)	1 (1.0)	0 (0.0)		1 (1.8)	0 (0.0)	
Other reason	10 (6.5)	6 (6.3)	4 (6.8)		3 (5.3)	7 (7.1)	
Unknown/missing	8 (5.2)	4 (4.2)	4 (6.8)		4 (7.0)	4 (4.1)	

^1^ Patients received carboplatin + pemetrexed (*n* = 8), carboplatin + paclitaxel (*n* = 6), cisplatin + pemetrexed (*n* = 3); ^2^ Patients received bevacizumab + carboplatin + paclitaxel (*n* = 10), bevacizumab + carboplatin + pemetrexed (*n* = 3); ^3^ Other regimens were any treatments that were not predefined selections during chart review; ^4^ Patients received carboplatin + pemetrexed (*n* = 11), carboplatin + paclitaxel (*n* = 3), cisplatin + pemetrexed (*n* = 2); ^5^ Patients received bevacizumab + carboplatin + paclitaxel (*n* = 2); ^6^ Other regimens were any treatments that were not predefined selections during chart review or patients involved in clinical trials; ^7^ Percentages for reasons of discontinuation of crizotinib therapy are out of the number of patients with a known end date. Patients without a known end date were considered to be in an ongoing therapy. ^†^ The focus of the study was to assess crizotinib treatment by 1 L or ≥2 L however it was important to capture those treatments occurring prior to and after crizotinib treatment to better understand the treatment landscape in this population. ^±^ Each percentage is calculated for specific treatment received out of those patients receiving pre or post crizotinib treatment.

**Table 3:** Healthcare resource utilization during crizotinib treatment (reported on a per month basis).

	Overall (*n* = 199)	Line of Therapy for Crizotinib Initiation		
		First-Line (*n* = 123)	Second/Later-Line (*n* = 76)	*p*-Value
**Visits to an emergency room (on outpatient basis) ^1^**				
Had ≥ 1 visit, *n* (%)	28 (14.1)	19 (15.5)	9 (11.8)	0.53
**Hospital admissions (overnight stay or day admission excluding ER visits) for reasons directly related to NSCLC ^1^**				
Had ≥ 1 admission, *n* (%)	61 (30.7)	41 (33.3)	20 (26.3)	0.34
**Outpatient visits**				
Had ≥ 1 claim, *n* (%)	170 (85.4)	104 (84.6)	66 (86.8)	0.84
Median Cost (min, max)	$108.65 (9.30, 336.88)	$110.50 (9.30, 334.99)	$99.38 (18.86, 336.88)	
**Laboratory procedures**				
Had ≥ 1 claim, *n* (%)	153 (76.9)	90 (73.2)	63 (82.9)	0.12
Median Cost (min, max)	$26.12 (1.08, 153.49)	$25.24 (1.08, 153.49)	$26.29 (2.32, 127.76)	
**Radiotherapy**				
Had ≥ 1 claim, *n* (%)	37 (18.6)	24 (19.5)	13 (17.1)	0.71
Median Cost (min, max)	$268.71 (7.63, 1613.79)	$276.09 (7.63, 1613.79)	$219.90 (54.65, 576.38)	
**Imaging ^2^**				
Had ≥ 1 claim, *n* (%)	53 (26.6)	31 (25.2)	22 (29.0)	0.76
Median Cost (min, max)	$69.84 (4.43, 206.86)	$64.17 (4.43, 180.72)	$71.72 (6.97, 206.86)	

^1^ Emergency visits and hospitalization costs could not be calculated as inpatient claims data were not captured. Utilization was captured from chart review. ^2^ Imaging procedures with a zero cost allowance in the 2014 Medicare data were counted as utilizations but not included in the counts for total cost.
